# Identification of hub genes and pathways in colitis-associated colon cancer by integrated bioinformatic analysis

**DOI:** 10.1186/s12863-022-01065-7

**Published:** 2022-06-22

**Authors:** Yongming Huang, Xiaoyuan Zhang, PengWang, Yansen Li, Jie Yao

**Affiliations:** 1 Department of General Surgery, Affiliated Hospital of Jining Medical University, 89 Guhuai Road, Jining, 272000 Shandong Province China; 2 Key Laboratory of Precision Oncology in Universities of Shandong, Department of Pathology and Institute of Precision Medicine, Taibai Lake New Area, Jining Medical University, 133 Hehua Road, Jining, 272067 Shandong Province China; 3 Department of Oncology, Jining Hospital of Traditional Chinese Medicine, 3 Huancheng North Road, Jining, 272000 Shandong Province China

**Keywords:** Colitis-associated colon cancer, Differentially expressed genes, Signaling pathways, functional enrichment analysis, Prognosis

## Abstract

**Background:**

Colitis-associated colon cancer (CAC) patients have a younger age of onset, more multiple lesions and invasive tumors than sporadic colon cancer patients. Early detection of CAC using endoscopy is challenging, and the incidence of septal colon cancer remains high. Therefore, identifying biomarkers that can predict the tumorigenesis of CAC is in urgent need.

**Results:**

A total of 275 DEGs were identified in CAC. *IGF1*, *BMP4*, *SPP1*, *APOB*, *CCND1*, *CD44*, *PTGS2*, *CFTR*, *BMP2*, *KLF4*, and *TLR2* were identified as hub DEGs, which were significantly enriched in the PI3K-Akt pathway, stem cell pluripotency regulation, focal adhesion, Hippo signaling, and AMPK signaling pathways. Sankey diagram showed that the genes of both the PI3K-AKT signaling and focal adhesion pathways were upregulated (e.g., *SPP1*, *CD44*, *TLR2*, *CCND1*, and *IGF1*), and upregulated genes were predicted to be regulated by the crucial miRNAs: hsa-mir-16-5p, hsa-mir-1-3p, et al. Hub gene-TFs network revealed FOXC1 as a core transcription factor. In ulcerative colitis (UC) patients, *KLF4, CFTR, BMP2, TLR2* showed significantly lower expression in UC-associated cancer. *BMP4* and *IGF1* showed higher expression in UC-Ca compared to nonneoplastic mucosa. Survival analysis showed that the differential expression of *SPP1*, *CFRT*, and *KLF4* were associated with poor prognosis in colon cancer.

**Conclusion:**

Our study provides novel insights into the mechanism underlying the development of CAC. The hub genes and signaling pathways may contribute to the prevention, diagnosis and treatment of CAC.

**Supplementary Information:**

The online version contains supplementary material available at 10.1186/s12863-022-01065-7.

## Introduction

Colon cancer is the third leading cause of cancer-associated death worldwide. Sporadic, hereditary, and colitis-associated colon cancer (CAC) are the three categories of this disease based on etiology. CAC is a major complication of inflammatory bowel disease (IBD). Compared with the age- and sex-matched general population, patients with IBD have a twofold increased risk of developing colon cancer [1]. Owing to a rising incidence and duration of IBD, the prevalence of CAC has also increased. Previously published epidemiological data has shown that the incidence of CAC ranges from 0.64% to 0.87% among the general population. However, 8%–16% of these patients die of the disease [2–4]. In terms of clinical features, CAC patients have a younger age of onset and more multiple lesions and invasive tumors than sporadic colorectal cancer patients; in addition, the prognosis of these patients is poor [5]. Early detection of CAC using endoscopy is challenging, and the incidence of septal colon cancer remains high. Thus, the discovery of specific molecular markers for CAC is urgently required.

It is widely known that microarray and RNA sequencing are both primary techniques used in transcriptome analysis. Horever, microarray is the common choice of most researchers since RNA-Seq is a expensive technique with data storing challenges and complex data analysis [6, 7]. Microarrays have widely been used to explore and identify the specific biomarkers for diagnosis and prognosis of disease [8]. Previously, bioinformatics analyses of CAC were mainly conducted by using gene chips of ulcerative colitis and colon adenocarcinoma [9, 10]. However, not all patients with ulcerative colitis would develop colon cancer. Meanwhile, some studies have demonstrated that there were significant changes in genome-wide RNA patterns between sporadic colon cancer and CAC patients [11]. Therefore, as the genes involved in the development of CAC and the relationship between those genes is still unclear [12], it is imperative to explore and reveal the accurate genes and signaling pathways of CAC.

In this study, we downloaded GSE43338 and GSE44904 datasets from the publicly available Gene Expression Omnibus (GEO) database and normalized the data to identify the differentially expressed genes (DEGs) between CAC and normal adjacent (control) tissues. In addition, this study provides a multi-level bioinformatics analysis strategy for identifying DEGs that consists of modular analysis, functional enrichment analysis, and screening of core genes by constructing a protein–protein interaction network (PPI) and the Sankey diagram of core genes. Gene-related network analyses were performed using NetworkAnalyst. The mRNA expression of hub genes were examined in ulcerative colitis-associated cancer patients. Prognostic analysis of hub genes was conducted based on The Cancer Genome Atlas (TCGA) data. Our findings may contribute to a better understanding of the mechanisms underlying the occurrence and development of CAC.

## Material and methods

### Acquisition and processing of gene expression set

GSE44904 and GSE43338 datasets were downloaded from the GEO database (Gene Expression Omnibus, https://www.ncbi.nlm.nih.gov/geo). The platform for the dataset GSE44904 is GPL7202 (Agilent-014868 Whole Mouse Genome Microarray 4 × 44 K G4122), which includes the AOM/DSS group (*n* = 3), DSS group (*n* = 3), AOM group (*n* = 3), and control group (*n* = 3). The platform for dataset GSE43338 was GPL339 ([MOE430A] Affymetrix Mouse Expression 430A Array). The CAC group (*n* = 4) and CAC control group(*n* = 2) were selected as per the needs of the study. The R software limma package Version 4.0, (http://www.bioconductor.org/) [13] was used to calibrate the data, the platform annotation file was used to annotate the probe, and the probe that did not match the gene (gene symbol) was removed. In addition, for multiple probes mapped to the same gene, the average value was calculated as the final expression value.

### Screening and VENN analysis of DEGs

Two or more groups of samples were compared using the limma R package, and the genes with adj. *P*. Val < 0.05 and |log fold change (FC)|> 2 were considered to be DEGs. The upregulated and downregulated gene lists were saved as Excel files, and the TXT files of all gene lists sorted by logFC in each dataset were saved for subsequent analysis. The bioinformatics online tool (AIPuFu, www.aipufu.com) was used to analyze the data obtained by VENN. The DEGs in the GSE44904 dataset were screened by VENN to identify the differential genes expressed alone in the AOM/DSS group. Then, above differential genes intersecting with the upregulated and downregulated DEGs of GSE43338 dataset were used as the target DEGs for follow-up analysis.

### Construction of PPI protein interaction network and module analysis

The Search Tool for the Retrieval of Interacting Genes (STRING, https://cn.string-db.org/) is an online database that explores functional interactions between proteins encoded by differential genes and visualizes the PPI-protein interaction network of DEGs [14]. We selected the PPI relation pairs with a combined score > 0.4, eliminated the scattered PPI pairs, and mapped them to the network. The PPI network diagram was constructed using the Cytoscape software (https://cytoscape.org/). The MCODE plugin in the Cytoscape software was used to filter the submodules based on the default parameters "Degree Cutoff = 2″, "Node Score Cutoff = 0.2″, "K-Core = 2″ and " Max. Depth = 100".

### Screening of hub genes for DEGs

The Cytohubba plug in the Cytoscape software was used to screen hub genes. TOP 15 nodes were calculated by Degree, Closeness and Radiality methods in Cytohubba. Scores were calculated by the Cytohubba plugin, and the top 11 genes with the most significance in the survival analysis were selected as hub genes according to their score.

### Functional enrichment analysis of genes

The database used for annotation, visualization, and integrated discovery (DAVID, http://david.ncifcrf.gov/) is an online tool that provides a comprehensive set of functional annotation methods for a range of genes or proteins provided by researchers [15]. The identified genes were analyzed for GO annotation and KEGG (https://www.kegg.jp/kegg/kegg1.html) pathway enrichment using the DAVID tool. *P* < 0.05 was selected as the threshold for considering genes to be enriched, and the TXT file of the above analysis results was downloaded for further analysis.

### Analysis of transcriptional factors (TFs) and miRNAs of hub genes

NetworkAnalyst3.0 (https://www.networkanalyst.ca) is a comprehensive network visual analysis platform for gene expression analysis and meta- analysis [16]. JASPAR database on the platform was used to analyze the TFs related to the hub genes. The gene-miRNA target interaction network was built using the miRNet 2.0.

### mRNA expression of hub genes were examined in patients

Microarray mRNA expression data of GSE3629 was taken from GEO. All statistical analyses and plots were conducted using R software. Shapiro–Wilk normality test and Wilcoxon rank-sum test were used to analyze the expression of hub genes in UC-Ca and UC-NonCa samples, respectively [17].

### Survival analysis of hub genes

The survival analysis of the identified hub genes was carried out by using the online software UALCAN (http://ualcan.path.uab.edu/index.html), which uses TCGA Level 3 RNA-seq and clinical data from 31 cancer types. UALCAN can estimate the effect of gene expression levels and clinicopathologic features on patient survival [18].

## Results

### Microarray data normalization and identification of DEGs

**Fig. 1:**
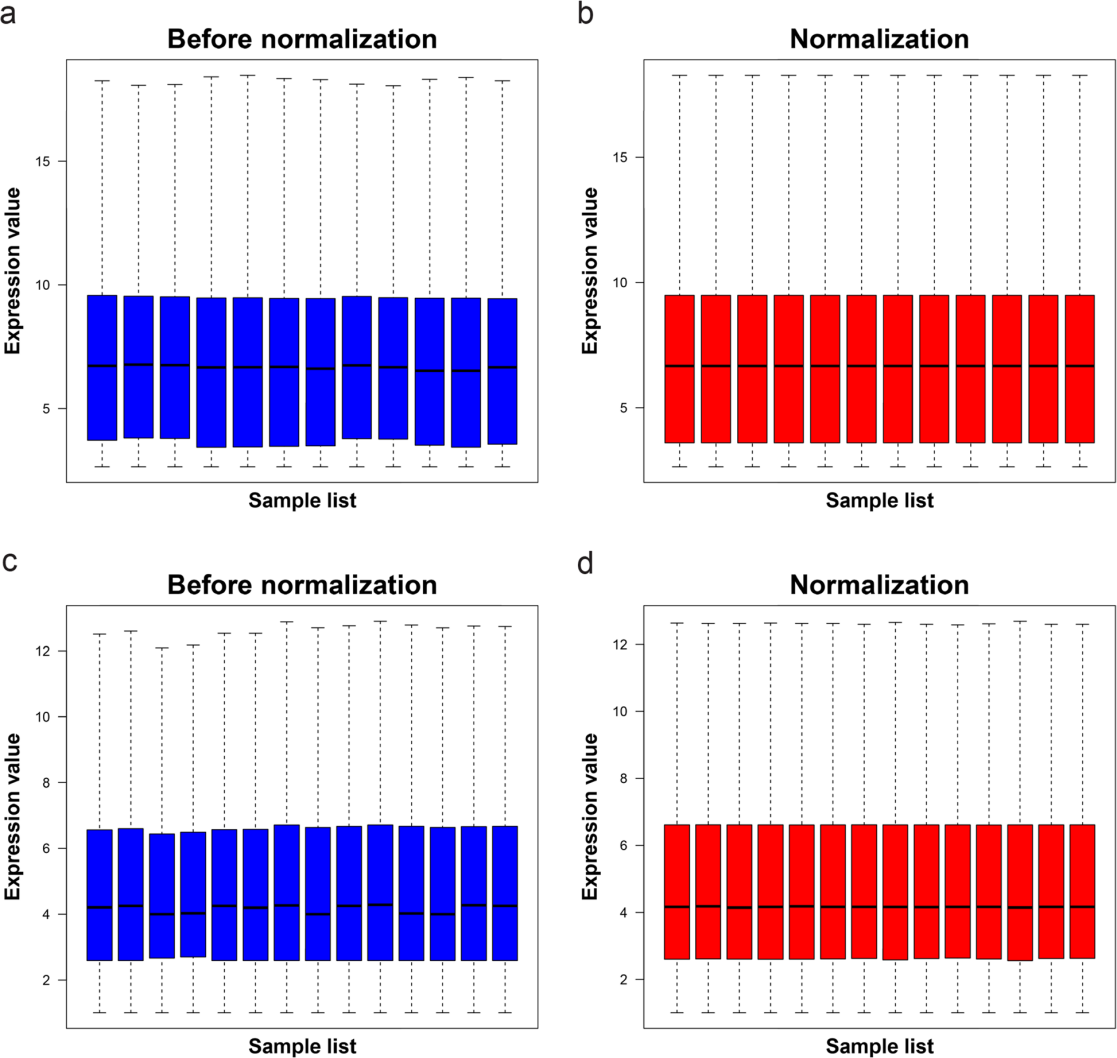
Normalized gene expression. The normalization of GSE44904 dataset (**a** and **b**). The normalization of GSE43338 dataset (**c** and **d**). Blue represents data before normalization, and red represents data after normalization

**Fig. 2:**
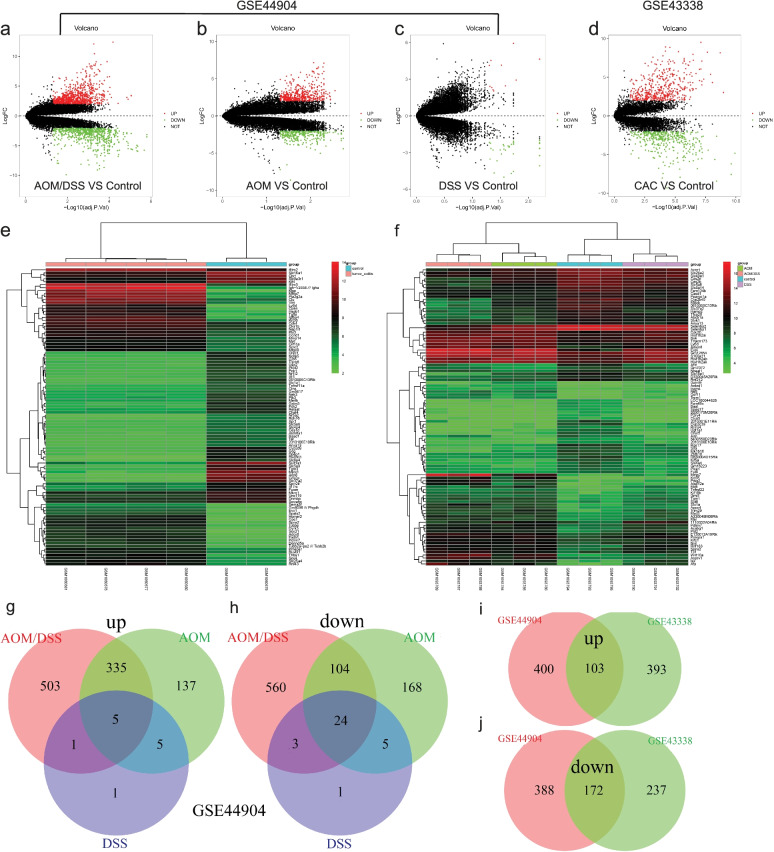
Identification of DEGs from two dataset chips. Different groups in GSE44904 dataset: AOM/DSS VS Control group (**a**), AOM VS Control group (**b**), DSS VS Control group (**c**), and (**d**) GSE43338 dataset (CAC VS Control group). adj. P. Val < 0.05 and | log a fold change |< 2, red dots represent upregulated genes, green dots represent downregulated genes, and black dots represent genes with no significant difference. Heat maps of the top 100 DEGs in GSE44904 (**e**) and GSE43338 (**f**) datasets. Red indicates relative upregulation of gene expression; green indicates relative downregulation of gene expression. VENN diagram of DEGs identified from datasets (g&h: DEGs were only expressed in the AOM/DSS group from GSE44904 dataset; i&j: overlapping DEGs which were upregulated and downregulated in the two datasets)

The chip expression datasets GSE44904 and GSE43338 were normalized, and the results are shown in Fig. 1. The limma R package (adjusted *p* < 0.05, and | log fold change (fc) |> 2) was used to screen DEGs. First, different groups in GSE44904 were compared, the different volcanoes plots are shown in Fig. 2a- c. Second, a total of 905 DEGs, comprising 496 upregulated and 409 downregulated genes, were screened from the dataset GSE43338. The DEGs of GSE43338 datasets are shown in Fig. 2d**.** A heat map was drawn for the top 100 DEGs as shown in Fig. 2e&f. Based on the different groups in the GSE44904 dataset, we further performed Venn analysis to screen out DEGs solely in CAC. Then a total of 1063 DEGs were identified, comprising 503 upregulated and 560 downregulated genes (Fig. 2g-h). Based on the DEGs screened from the two data sets, a Venn analysis was repeated, and 275 overlapping genes were found, comprising 103 upregulated and 172 downregulated genes (Fig. 2i-j).

### PPI network construction and functional analysis of DEGs

**Fig. 3:**
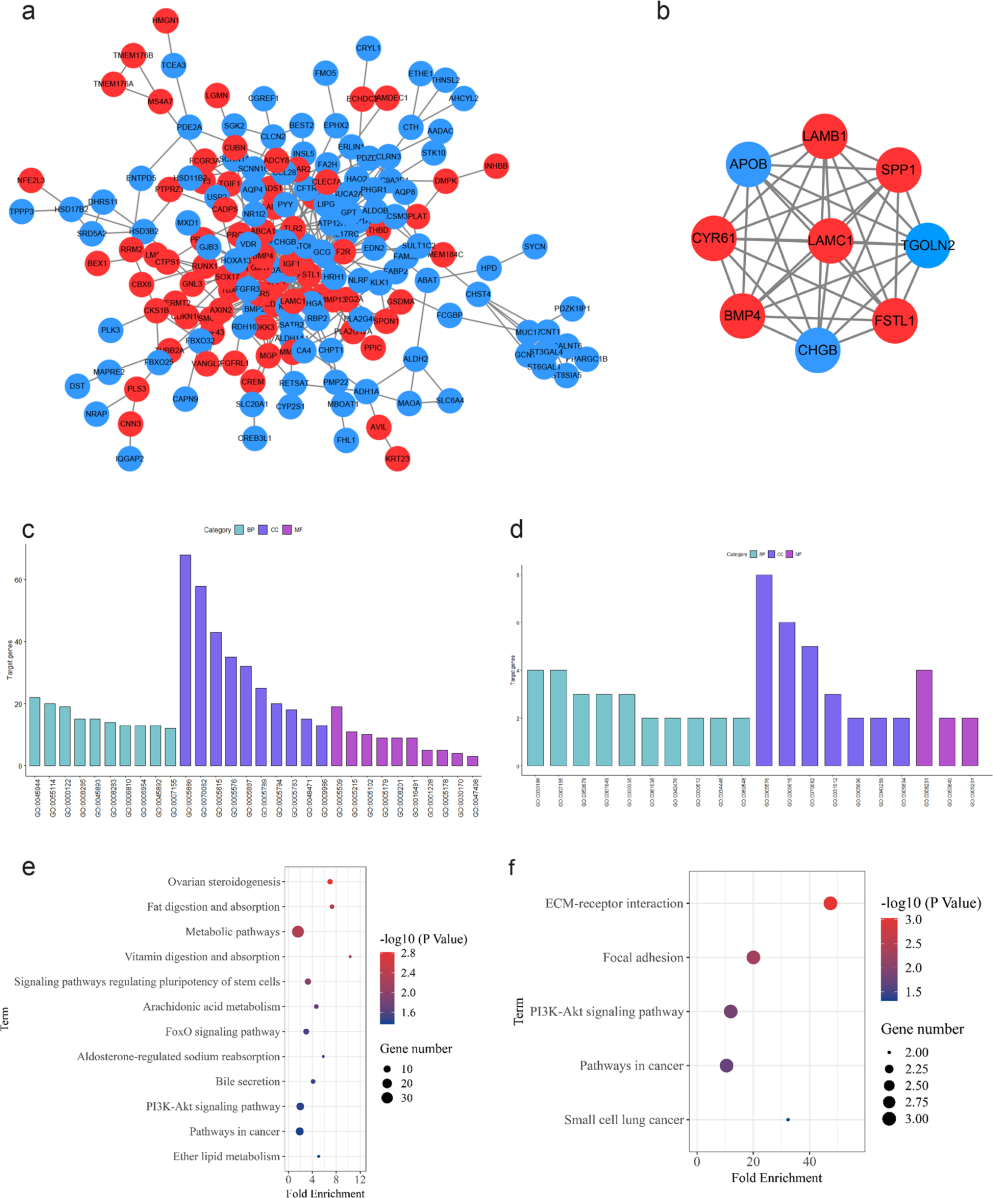
Protein–protein network and module analysis of DEGs. The network map of DEGs was constructed using STRING (**a**). The modular analysis was carried out on the network to screen out the module (**b**) with the highest score (MCODE score = 9.0). Red represents upregulated genes and the blue represents downregulated genes. Gene ontology (GO) enrichment analysis in DEGs and module genes were performed using the DAVID Database (c: DEGs, d: module genes); Classification: Biological Process (BP), B: Cellular Component (CC), C: Molecular Function (MF). KEGG pathways using the ggplot2 package in R language for visualization (e: DEGs, f: module genes). The size of the dot represents the amount of gene enrichment, and the color of the dot represents *p* value

The STRING online database was used to analyze the 275 intersecting DEGs. A PPI network was constructed as shown in Fig. 3a. To study the functional annotation of the selected DEGs, DAVID analysis was performed to categorize genes by biological process (BP), molecular function (MF), and cellular component (CC). The results were considered statistically significant at *p* < 0.05; the GO results are shown in Fig. 3c. BP mainly includes positive regulation of transcription from RNA polymerase II promoter, oxidation–reduction process, negative regulation of transcription from RNA polymerase II promoter, negative regulation of cell proliferation, positive regulation of transcription, DNA-templated, cell proliferation, transport, inflammatory response, negative regulation of transcription, DNA-templated, cell adhesion, among others. CC mainly includes extracellular space, plasma membrane, extracellular exosome, extracellular region, integral component of plasma membrane, endoplasmic reticulum membrane, Golgi apparatus, endoplasmic reticulum, and others. MF mainly includes hormone activity, transporter activity, calcium ion binding, receptor binding, heparin binding, and oxidoreductase activity. We performed KEGG analysis of DEGs and as shown in Fig. 3e, the pathways mainly enriched were ovarian steroidogenesis, fat digestion and absorption, metabolism, vitamin digestion and absorption, and regulation of pluripotency of stem cells, arachidonic acid metabolism, FoxO signaling pathway, aldosterone-regulated sodium reabsorption, bile secretion, PI3K-Akt pathway, cancer, and ether lipid metabolism.

To further understand the DEGs, the MCODE plugin in the Cytoscape software was subsequently used for modular analysis, and the sub-modules with high scores were selected with a score of 9. Module genes were *SPP1*, *Tgoln2*, *ApoB*, *FSTL1*, *LAMB1*, *LAMC1*, *CHGB*, *BMP4*, and *CYR61* (Fig. 3b). The GO function analysis results for the submodule genes are shown in Fig. 3d. BP mainly includes extracellular matrix organization, cell adhesion, positive regulation of epithelial cell proliferation, and positive regulation of cell migration. CP mainly includes the extracellular region, extracellular space, and extracellular exosomes. MF mainly includes heparin binding and extracellular matrix binding. KEGG pathway analysis showed that genes were mainly enriched in ECM-receptor interaction, focal adhesion, PI3K-Akt signaling pathway, and cancer pathways, such as small cell lung cancer pathways (Fig. 3f).

### Hub genes selection and analysis

**Fig. 4:**
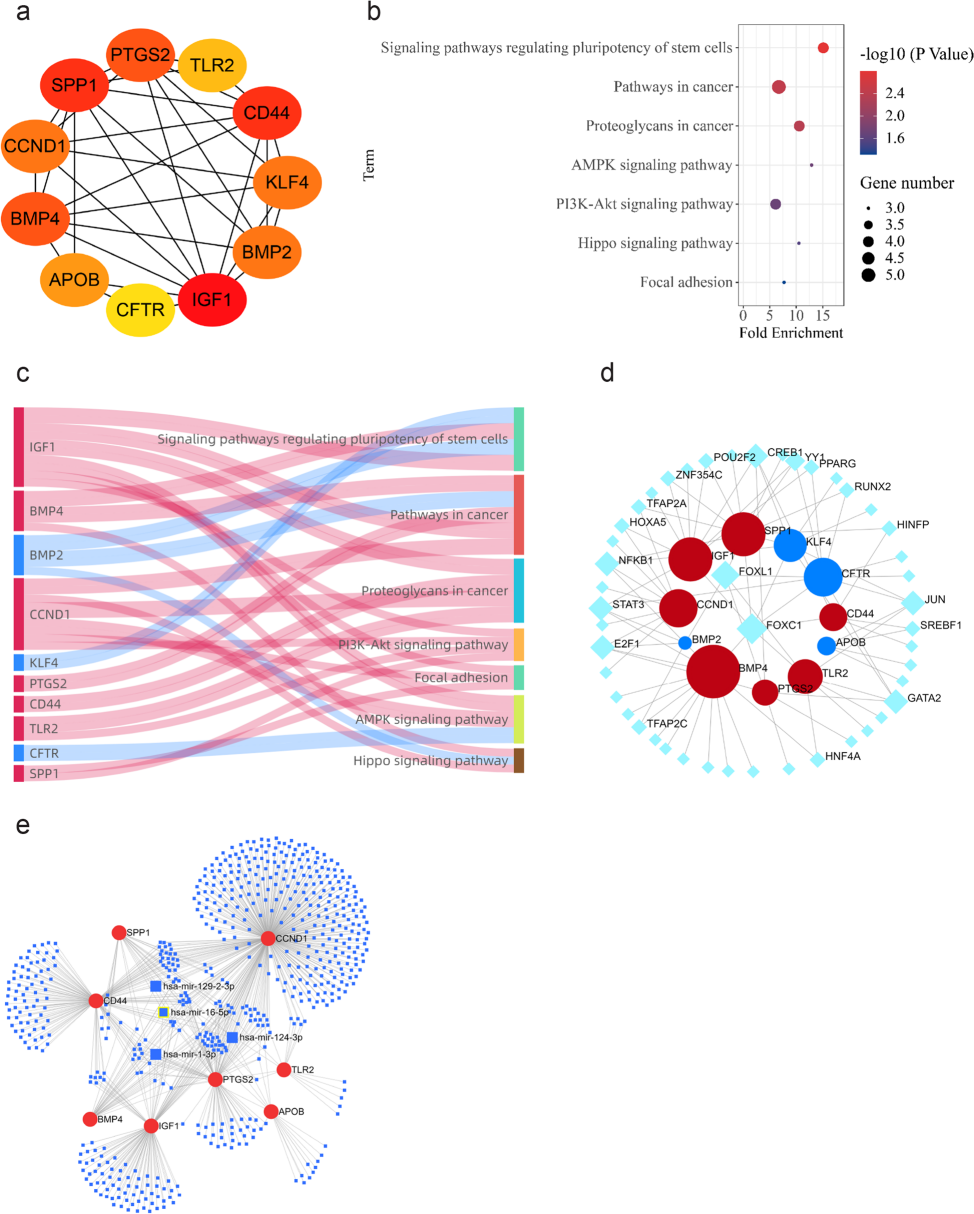
The hub genes were screened and analyzed by KEGG and correlation analysis. The top 11 genes with the most significance were selected as hub genes according to the score (**a**). KEGG pathway analysis of hub genes was analyzed by DAVID (**b**). The distribution relationship between hub genes and pathways (**c**): Red represents upregulated genes and blue represents downregulated genes. Correlation analysis of core TF and hub genes (**d**) and gene-miRNA interactions network (**e**), circles represents genes, diamonds represents TFs, and squares represents the miRNAs, sizes represents the degree

The scores of DEGs were calculated using the Cytoscape software, and the top 11 genes were selected as hub genes (Fig. 4a). These included *IGF1, BMP4, SPP1, APOB, CCND1, CD44, PTGS2, CFTR, BMP2, KLF4, and TLR2*. Detailed information on the hub genes, is shown in Table 1. The scores calculated by the Radiality and Closeness methods in the cytohubba pluginto were shown in Table S1. To determine the enriched pathways terms for hub genes, KEGG pathway analysis was performed using DAVID. The genes were enriched in signaling pathways regulating many biological functions (Fig. 4b). The Sankey diagram shows the distribution of hub genes in the different signaling pathways (Fig. 4c): signaling pathways regulating pluripotency of stem cells (enriched genes: *IGF1*, *BMP4*, *BMP2*, *KLF4*; *p* = 0.0015), pathways in cancer (enriched genes: *BMP4*, *BMP2, CCND1, IGF1*, and *PTGS2*; p = 0.0035), proteoglycans in cancer (enriched genes: *CCND1*, *IGF1, CD44*, and *TLR2*; *p* = 0.0043), AMPK signaling pathway (enriched genes: *CCND1, IGF1, CFTR*; *p* = 0.0186), PI3K-Akt signaling pathway (enriched genes: *CCND1, SPP1, IGF1, TLR2*; *p* = 0.0196), Hippo signaling pathway (enriched genes: *BMP4, BMP2, CCND1*; *p* = 0.0273), and pathways involved in focal adhesion (enriched genes: *CCND1, SPP1, IGF1*; *p* = 0.0483).

**Table1:** Detailed information about the hub gene

Gene symbols	Type	Degree	Full name	Encoded protein function
*IGF1*	up	24	Insulin-like growth factor 1	The encoded protein is a member of a family of proteins involved in mediating growth and development
*BMP4*	up	23	Bone morphogenetic protein 4	The encoded protein is possibly involved in the pathology of multiple cardiovascular diseases and human cancers
*SPP1*	up	22	Secreted phosphoprotein 1	The encoded protein is a cytokine that upregulates the expression of interferon-ɣ and interleukin-12
*APOB*	down	22	Apolipoprotein B	The encoded protein affects plasma cholesterol and apolipoprotein levels in various diseases
*CCND1*	up	20	Cyclin D1	The encoded protein alters cell cycle progression, and its expression is widely observed in various human cancers
*CD44*	up	18	CD44 molecule	The encoded protein participates in various cellular functions, including lymphocyte activation, recirculation, and homing; hematopoiesis; and tumor metastasis
*PTGS2*	up	18	Prostaglandin-endoperoxide synthase 2	The encoded protein is responsible for activating prostanoid biosynthesis involved in inflammation and mitogenesis
*CFTR*	down	16	CF transmembrane conductance regulator	The encoded protein acts as a chloride channel, and it controls ion and water secretion and absorption in epithelial tissues
*BMP2*	down	16	Bone morphogenetic protein 2	The encoded protein plays a role in bone and cartilage development
*KLF4*	down	14	Kruppel-like factor 4	The encoded protein controls the G1-to-S transition of the cell cycle following DNA damage by mediating the expression of the tumor suppressor gene *p53*
*TLR2*	up	14	Toll-like receptor 2	The encoded protein regulates host inflammation and promotes apoptosis in response to exposure to bacterial lipoproteins

The TF-gene regulatory network was constructed based on the JASPAR database on the Network Analyst platform. Figure 4d depicts the transcription factors that can regulate two or more genes. In addition to hub genes, there were 46 transcription factors in the regulatory network, and 86 relationship pairs were established. Among the predicted transcription factors, FOXC1 is considered to be the core TF that can regulate multiple genes, including *SPP1, IGF1, BMP4, TLR2, CD44, KLF4,* and *CFTR*. In order to further investigate the upregulated genes in the hub genes, we performed gene-miRNA interactions network using miRNet 2.0. A total of 8 genes, 613 miRNAs, and 823 gene-miRNA pairs were registered in the network (Fig. 4e). Main miRNAs with interactions of more than six genes are listed in Table S2. It was predicted that hsa-miR-16-5p could regulate *CCND1, CD44, PTGS2, IGF1, APOB, SPP1,* and *BMP4*, while hsa-miR-1-3p could regulate *CCND1, CD44, IGF1, PTGS2, APOB,* and *BMP4*.

### mRNA expression of the hub genes in patients

**Fig. 5:**
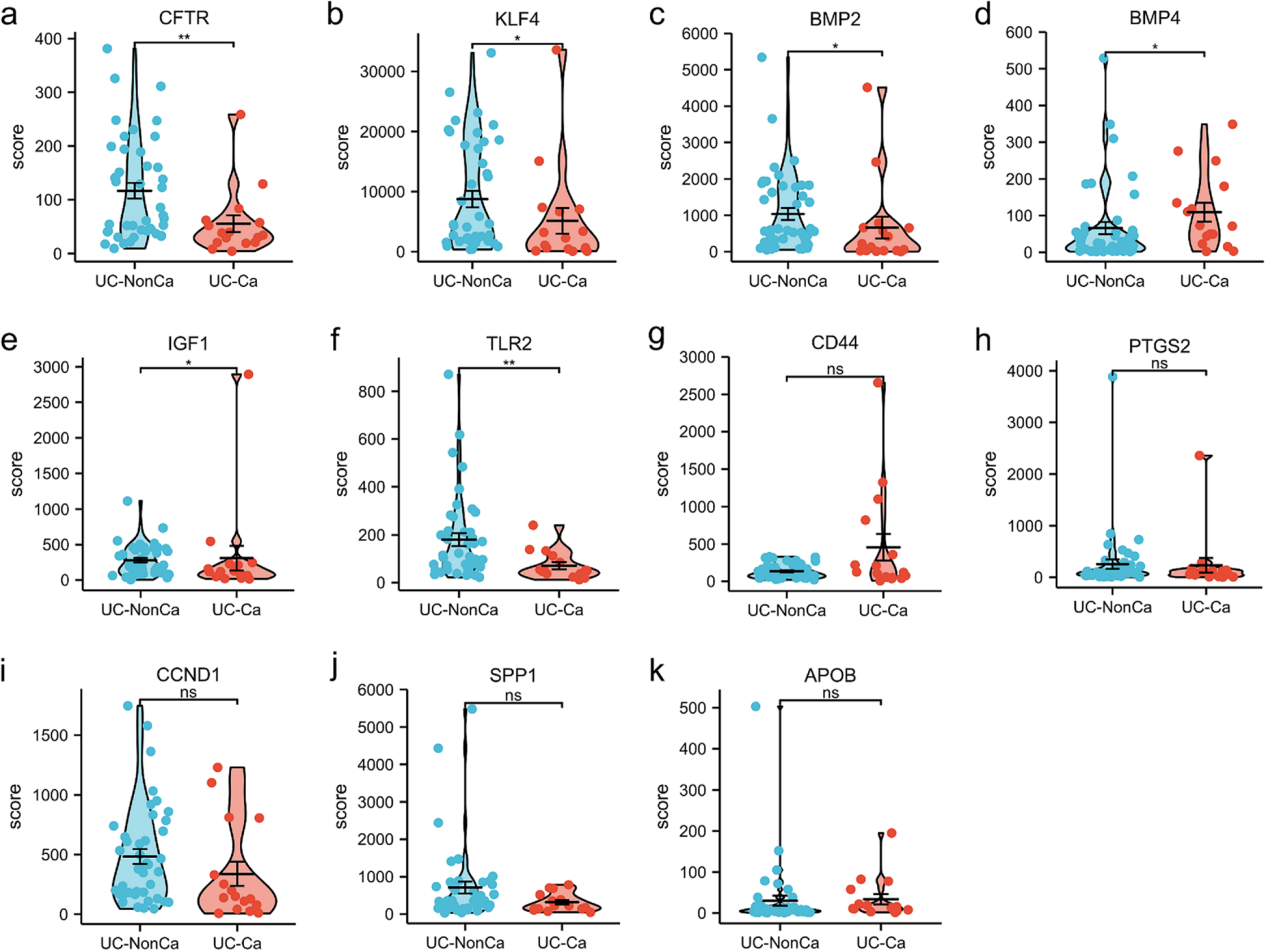
The mRNA expression level of hub genes in patients according to the GEO database. UC-NonCa indicates nonneoplastic mucosa tissue of ulcerative colitis patients, and UC-Ca indicates ulcerative colitis-associated cancer tissue. ns, *p* ≥ 0.05; *, *p* < 0.05; **, *p* < 0.01; ***, *p* < 0.001

mRNA expression results of hub genes in the GSE3629 indicated that *CFTR*(*p* < 0.01)*, KLF4*(*p* < 0.05), *BMP2*(*p* < 0.05) and *TLR2*(*p* < 0.01) were downregulated. *BMP4*(*p* < 0.05), and *IGF1*(*p* < 0.05) were upregulated. These were consistent with our analysis results. There were no significant differences in mRNA expression of *CD44*, *PTGS2*, *CCND1*, *SPP1* and *APOB* (Fig. 5).

### Survival analysis of hub genes in colon cancer

**Fig. 6:**
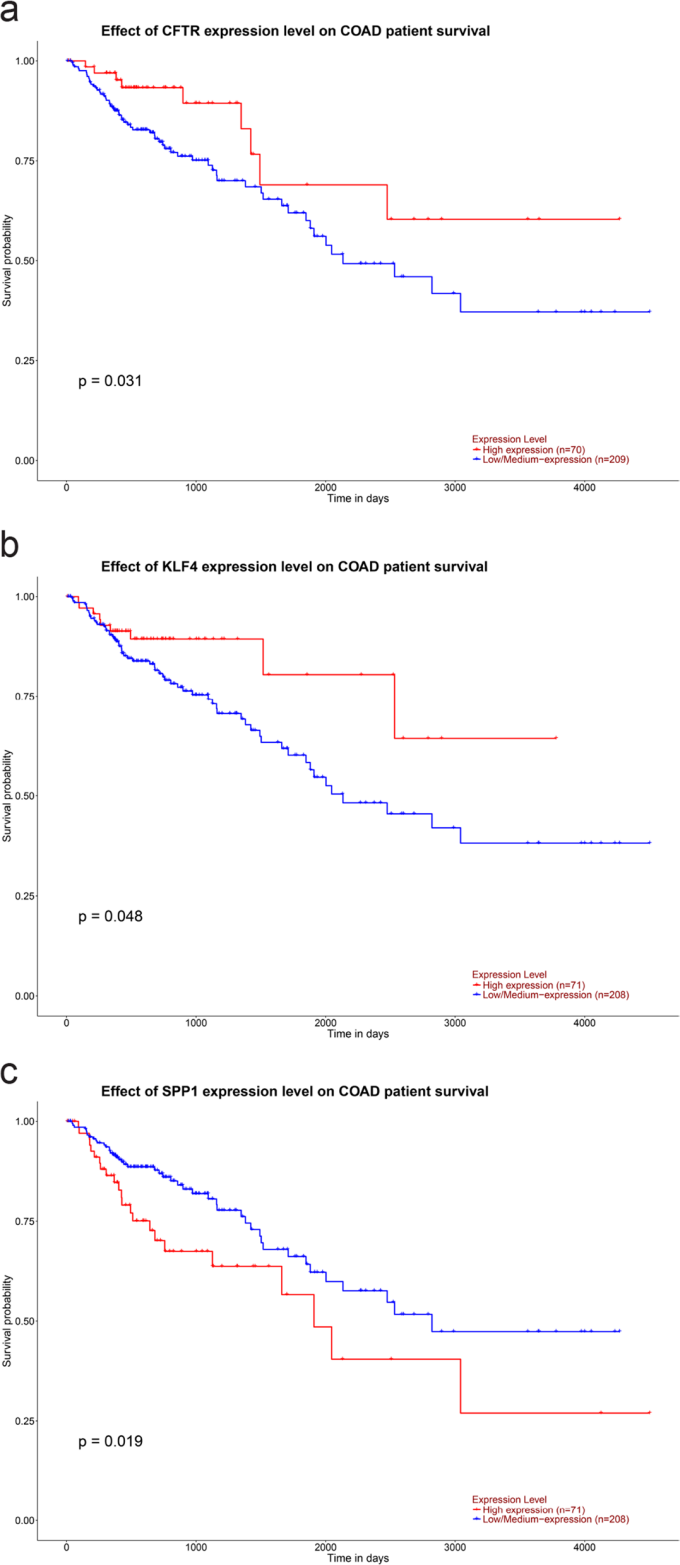
Survival analysis of hub genes in colon cancer (*P* < 0.05). (a) CFTR, (b) KLF4, (C) SPP1

Considering CAC as an etiological classification of colon cancer, we used colon cancer data from the TCGA database to analyze the survival of hub genes (Fig. 6). Survival analysis data contained information on high or low expression of target genes, as well as that on the correlation between hub genes and colon cancer. Among the 11 hub genes, the following genes were found to be associated with the prognosis of colon cancer patients: *SPP1* (*p* = 0.019), *CFTR* (*p* = 0.031), and *KLF4* (*p* = 0.048).

## Discussion

Not all patients with inflammatory bowel disease develop CAC. Therefore, comparing the differentially expressed genes in the CAC model and those in the IBD model may enable us to find specific genes in CAC. In this study, data from the GEO database (GSE44904 and GSE43338) were normalized, different groups of the GSE44904 dataset were analyzed. Through Venn analysis, DEGs alone in CAC (AOM/DSS) were screened. Through intersection analysis using gene microarray data from the CAC animal model in the GSE43338 dataset, a total of 275 specific genes (including 103 upregulated and 172 downregulated genes) were found in CAC. GO and KEGG pathway analyses of the selected DEGs indicated that some biological processes and functions were associated with CAC, such as regulation of transcription from RNA polymerase II promoter, reduction process, cell proliferation, inflammatory response, cell adhesion, extracellular space, plasma membrane, extracellular exosome, transporter activity, calcium ion binding, and receptor binding. Furthermore, the enrichment results of the genes in the submodules with the highest scores also confirmed the importance of these biological processes and functions. In the KEGG pathway analysis, a large number of differential genes were found to be enriched in metabolic pathways, which is consistent with published studies [19]. Lu and Wang, through metabonomics analysis, found that there were many metabolic pathway changes in colon cancer induced by AOM/DSS [20]. Our study also demonstrated that fat digestion and absorption, ovarian steroidogenesis, vitamin digestion and absorption, arachidonic acid metabolism, ether lipid metabolism, and other metabolic pathways are closely related to the occurrence and development of CAC.

However, interestingly, in addition to the metabolic pathway, a large number of DEGs were enriched in pathways in cancer, signaling pathways regulating pluripotency of stem cells, PI3K-Akt signaling pathway, and FoxO signaling pathway. Subsequently, KEGG pathway analysis was performed for the genes in the submodules. The pathways obtained were similar to those enriched in DEGs, such as the pathways involved in cancer, PI3K-Akt signaling pathway, and focal adhesion pathway. These results suggest that these pathways and their genes play key roles in the occurrence and development of CAC. Focal adhesion is the contact point between cells and the surrounding environment, which can drive cell migration. The signaling pathway plays an important role in wound healing and tumor metastasis. It has been found that low expression of miR-4728-3p in ulcerative colitis-associated colorectal cancer can influence *CAV1*, *THBS2*, and *COL1A2* genes as well as focal adhesion signaling, which is related to tumor pathogenesis [21]. Li and Wang found that activation of focal adhesion kinase prevented the development of ulcerative colitis and CAC [22].

Further, PPI network analysis was conducted on DEGs. According to the degree score value, we identified DEGs with the highest score and significance as hub genes, namely, *BMP4, SPP1, APOB, CCND1, CD44, PTGS2, CFTR, BMP2, KLF4, TLR2*, and *IGF1*. To validate the results of bioinformatics analysis, we examined the mRNA expression levels of hub genes in patients by using GEO databases. The results were basically consistent with the observed gene expression trends. There was no significant difference in mRNA expression of some hub genes, which may be due to the small sample size. KEGG pathway analysis for the hub genes revealed that these genes were not only enriched in signaling pathways regulating the pluripotency of stem cells, PI3K-Akt signaling pathway, and focal adhesion pathway, but also were enriched in the Hippo and AMPK signaling pathways. These genes and their enriched pathways are closely related to the occurrence and development of CAC. Pluripotency is a characteristic of stem cells, and a small number of cells in tumors have self-renewal ability and produce heterogeneous tumors [23]. P53 can inhibit the pluripotency of tumor stem cells. In a preclinical animal model of CAC, targeted knockout of stem cell-specific P53 was found to significantly increase tumor size and incidence [24]. Josse et al. also found that PI3K/Akt is the main pathway affected by the AOM/DSS model through miRNA chip experiments [25]. This finding is consistent with our findings. In human colon tissue infiltrated with inflammatory cells, the PI3K/Akt pathway is activated and mediates the progression of colitis and CAC through a positive feedback loop that maintains the recruitment of inflammatory cells [26].

In inflammation-related tumor models, inhibition of IGF1 signaling can reduce the number and size of colon tumors in wild-type mice [27]. IGF-1R knockout can activate the LKB1/AMPK pathway and play a protective role in colitis and CAC [28]. Chen et al. found that the Hippo pathway was involved in the occurrence of intestinal inflammation and progression of CAC in an experimental mouse model [29]. YAP1 is a transcriptional co-activator in the Hippo signaling pathway. PGE2 signaling can increase the expression and transcriptional activity of YAP1, and YAP1 further activates PTGS2 and PTGER4, which in turn can activate PGE2. This positive feedback loop plays an important role in colon regeneration and promotes the development of colitis-related cancer [30]. In a mouse model of CAC, Ya-Chun Chou demonstrated that Boswellia serrata mediated Akt/GSK3β/cyclin D1 signaling pathway and altered the composition of gut microbiota to alleviate tumor growth [31].

Furthermore, other hub genes were significantly associated with the development of CAC. For example, an abnormal expression of BMP protein is a common feature of cancer. In the colon mucosa, the BMP pathway overlaps with several other colon cancer pathways [32]. Inhibition of the BMP pathway is an early event in inflammation-driven colon tumors in mice [33]. TLR2 is highly expressed in tumor tissues of CRC patients. Gene knockout and knockdown of TLR2 can inhibit the proliferation of inflammation-related colorectal cancer and sporadic colorectal cancer [34]. SPP1 is an important inflammatory mediator. It is upregulated in inflammation-related intestinal tumors and mediates the progression of colon cancer [35]. Yang and Liu found that deletion of KLF4 causes genetic instability, which in turn lead to the progression of CAC [36]. The mutation of the APOB gene in CRC associated with ulcerative colitis was found by whole exon sequencing, and there was a significant difference between ulcerative colitis-associated CRC and scattered CRC [37]. CD44 is an adhesion and anti-apoptotic molecule that is highly expressed in colon cancer [38]. However, in a comparative study, CD44 expression was found to be lower in ulcerative colitis-associated dysplasia and cancers than in sporadic colonic tumors [39].

The regulatory network of TF-gene predicted analysis showed that FOXC1, FOXL1, NFKB1, STAT3, JUN, E2F1, CREB1, and GATA2 were significantly related to hub gene. Recent studies have emphasized the important role of transcription factor nuclear factor kappa B (NF-κB) and signal transducer and activator of transcription 3 (STAT3) in the progression of inflammation-associated cancer [40, 41]. Meanwhile, transcription factors JUN [42], E2F1 [43], and GATA2 [44] have been reported to be closely related to the occurrence and development of colitis-associated tumors. FoxC1, as a core transcription factor, interacts most closely with hub genes. FoxC1 belongs to the forkhead box (FOX) transcription factor family. Many studies have confirmed that at least 14 proteins in the FOX transcription factor family are closely related to the pathogenesis of CRC [45]. Currently, as a new cancer marker and therapeutic target, the regulatory role of FOXC1 in many types of cancer has been widely studied [46]. Future studies should focus on CAC.

## Conclusion

In summary, based on GSE44904 and GSE43338 datasets, bioinformatics analysis identified 275 DEGs in CAC, including 103 upregulated and 172 downregulated genes. IGF1, BMP4, SPP1, APOB, CCND1, CD44, PTGS2, CFTR, BMP2, KLF4, and TLR2 were hub proteins, which were mainly related to the PI3K-Akt signaling pathway, focal adhesion, Hippo signaling pathway, AMPK signaling pathway, and stem cell pluripotency regulation pathway. The expression of hub genes were examined in the patient samples. A study on the TF-gene regulatory network of hub genes showed that FOXC1 was the core transcription factor, and had the most interaction with hub genes. Additional work is needed to elucidate the underlying mechanisms behind these observations. Survival analysis showed that the differential expression of *SPP1*, *CFRT*, and *KLF4* were associated with poor prognosis in colon cancer. This study helps us further understand the mechanism of CAC progression.

## Supplementary Information

**Additional file 1: Table S1.** Top 15 in network ranked by Closeness method and top 15 in network ranked by Radiality method.

**Additional file 2: Table S2.** The main related miRNAs of upregulated genes in the hub genes.

## Data Availability

Data is available at TCGA and GEO database, accession numbers: GSE44904: https://www.ncbi.nlm.nih.gov/geo/query/acc.cgi?acc=GSE44904. GSE43338: https://www.ncbi.nlm.nih.gov/geo/query/acc.cgi?acc=GSE43338. GSE3629: https://www.ncbi.nlm.nih.gov/geo/query/acc.cgi?acc=GSE3629.
