# Supplementary material for: Identification of hub genes and pathways in colitis-associated colon cancer by integrated bioinformatic analysis
Source: BMC Genom Data. 2022 Jun 22;23:48. doi: 10.1186/s12863-022-01065-7 (PMC9219145; doi:10.1186/s12863-022-01065-7)
Supplement: Supplementary file 1 — Additional file 1: Table S1. Top 15 in network ranked by Closeness method and top 15 in network ranked by Radiality method. [file 12863_2022_1065_MOESM1_ESM.docx]

Table S1

Top 15 in network ranked by Closeness method

| Rank | Name | Score |
| --- | --- | --- |
| 1 | IGF1 | 84.91667 |
| 2 | APOB | 82.3 |
| 3 | SPP1 | 80.66667 |
| 4 | BMP4 | 79.86667 |
| 5 | CD44 | 77.85 |
| 6 | CCND1 | 77.6 |
| 7 | PTGS2 | 76.85 |
| 8 | GCG | 75.55 |
| 9 | GJA1 | 74.58333 |
| 10 | CFTR | 73.73333 |
| 11 | BMP2 | 73.25 |
| 12 | KLF4 | 73.06667 |
| 13 | TLR2 | 72.58333 |
| 14 | FSTL1 | 72.48333 |
| 15 | CDX2 | 71.3 |

Top 15 in network ranked by Radiality method

| Rank | Name | Score |
| --- | --- | --- |
| 1 | IGF1 | 7.844899 |
| 2 | APOB | 7.798962 |
| 3 | SPP1 | 7.671362 |
| 4 | CD44 | 7.650946 |
| 5 | BMP4 | 7.635633 |
| 6 | PTGS2 | 7.610113 |
| 7 | GCG | 7.584593 |
| 8 | CCND1 | 7.569281 |
| 9 | GJA1 | 7.533553 |
| 10 | GPT | 7.518241 |
| 11 | KLF4 | 7.497825 |
| 12 | CFTR | 7.477408 |
| 13 | FSTL1 | 7.4672 |
| 14 | TLR2 | 7.456992 |
| 15 | BMP2 | 7.451888 |
