# Supplementary material for: Identification of hub genes and pathways in colitis-associated colon cancer by integrated bioinformatic analysis
Source: BMC Genom Data. 2022 Jun 22;23:48. doi: 10.1186/s12863-022-01065-7 (PMC9219145; doi:10.1186/s12863-022-01065-7)
Supplement: Supplementary file 2 — Additional file 2: Table S2. The main related miRNAs of upregulated genes in the hub genes. [file 12863_2022_1065_MOESM2_ESM.docx]

Table S2

The main related miRNAs of upregulated genes in the hub genes

| miRNA | Genes | Count |
| --- | --- | --- |
| hsa-mir-16-5p | CCND1, CD44, PTGS2, IGF1, APOB, SPP1, BMP4 | 7 |
| hsa-mir-1-3p | CCND1, CD44, IGF1, PTGS2, APOB, BMP4 | 6 |
| hsa-mir-124-3p | CCND1, CD44, SPP1, PTGS2, APOB, BMP4 | 6 |
| hsa-mir-129-2-3p | CCND1, CD44, IGF1, PTGS2, TLR2, BMP4 | 6 |
